# How Can We Improve the Assessment and Indifferent Outcomes From Pelvic Organ Prolapse Management From Conservative and Surgical Therapies? ICI‐RS 2025

**DOI:** 10.1002/nau.70272

**Published:** 2026-03-26

**Authors:** Rohna Kearney, Adrian Wagg, Wendy Bower, Chris Chapple, Vik Khullar, Dudley Robinson, Annika Taithongchai, Philip Toozs Hobson, Alan Wein, Paul Abrams

**Affiliations:** ^1^ The Warrell Unit, Saint Mary's Hospital, Manchester University NHS Foundation Trust Manchester Academic Health Science Centre Manchester UK; ^2^ Division of Developmental Biology & Medicine, School of Medical Sciences University of Manchester Manchester UK; ^3^ Department of Medicine, Division of Geriatric Medicine, College of Health Sciences Faculty of Medicine & Dentistry Edmonton Alberta Canada; ^4^ Continence Clinic and Department of Physiotherapy, The Royal Melbourne Hospital and Faculty of Medicine, Dentistry and Health Sciences The University of Melbourne Melbourne Australia; ^5^ Department of Urology, Sheffield Teaching Hospitals NHS Foundation Trust, Faculty of Health University of Sheffield Northern Ireland UK; ^6^ Department of Urogynaecology, St. Mary's Hospital Imperial College London UK; ^7^ Kings College Hospital London UK; ^8^ Department of Urogynaecology, Birmingham Women's and Children's Hospital NHS Foundation Trust Birmingham UK; ^9^ Desai Sethi Institute of Urology University of Miami Miller School of Medicine Miami Florida USA; ^10^ Bristol Urological Institute Southmead Hospital Bristol UK

**Keywords:** pelvic floor muscle training, pelvic organ prolapse recurrence, surgical failure, vaginal pessary

## Abstract

**Introduction:**

Pelvic organ prolapse is a common condition and many women seek surgical treatment for prolapse symptoms. However, recurrence of prolapse after surgical treatment is common. A think tank was held at ICI‐RS 2025 to discuss how the assessment and indifferent results from conservative and surgical management can be improved.

**Methods:**

Data were collected and presented on identification of women at risk of prolapse recurrence after surgery, understanding patient goals and expectations, optimising lifestyle interventions, pelvic floor muscle training, pessary management, and surgical care. Discussions identified knowledge gaps and proposed research studies that could advance knowledge to improve treatment outcomes.

**Results:**

There is insufficient information to understand the assessment of prolapse treatment outcomes; examination findings do not necessarily correlate with symptoms. Further research is needed to understand if patient‐reported goal attainment is superior to patient‐reported outcome measures, including measures of patient satisfaction. There is insufficient information on the value of lifestyle adjustments and pelvic floor muscle training as prehabilitation to improve surgical outcomes. The place of pessary management in an optimally integrated prolapse treatment pathway is unclear and the role that pessaries may have in anatomical modelling of prolapse is not fully understood. Further research into adjuncts to improve native tissue repair as alternatives to polypropylene mesh is needed to optimise surgical outcome.

**Conclusion:**

Further research into understanding what represents treatment, cure, and optimising conservative and surgical treatments is of high priority to improve pelvic organ prolapse treatment outcomes. The utility of preoperative rehabilitation requires investigation. Developing an optimised pessary care pathway and continued surgical innovation are required to ensure progress in reducing prolapse symptom recurrence.

## 1 Introduction

This topic was chosen by the members of the ICI‐RS for discussion at the 2025 meeting, given the perceived poor outcomes of pelvic organ prolapse (POP) surgery. The working group considered what factors needed to be addressed to improve outcomes for women with POP and, in particular, what treatments, such as vaginal pessaries, should be routinely offered to women.

POP is the most common pelvic floor disorder for which women seek surgical treatment. A 2011 Scottish registry linkage study of 34 651 women reported the lifetime risk of a woman undergoing prolapse surgery by the age of 80 was 9.5% with a re‐operation rate of 15.8% and a median of 3 years between index and repeat surgery [1]. A similar Australian study from 2010 reported a higher lifetime risk of prolapse surgery of 19% [2]. A US database study reported a lifetime prolapse surgery risk of 12.6% [3]. The reported reoperation rates are likely to underestimate the true incidence of surgical failure or symptom recurrence as many women will not seek further treatment or will pursue conservative management after an unsuccessful surgical procedure. The time at which recurrent symptoms present after index surgery may be a factor influencing further treatment choices.

For many years, the high postoperative recurrence rate of prolapse symptoms has been a concern of women and clinicians. The use of synthetic mesh was introduced in an attempt to reduce recurrence, but this has not had the desired effect and has been associated with significant complications. For example, the randomised PROSPECT study, comparing the use of native tissue, biological xenograft, and polypropylene mesh surgery in 1348 women with vaginal prolapse, reported at 4 and 6 years that the prolapse symptom score was better in women who had native surgery compared to polypropylene mesh [4]. Using a composite outcome assessing functional cure, half of the women were reported as not cured but only 10%–12% had undergone further prolapse surgery. In addition, 8.4% of women in the polypropylene mesh group had undergone further surgery for mesh‐related complications.

The true prevalence of POP symptoms is unknown and there are few data on how many women are undergoing conservative management for prolapse symptoms.

In 2025, the IC‐RS group discussed how to improve the assessment of prolapse and the indifferent results from conservative and surgical management. Topics of discussion included the identification of women at higher risk of failure and recurrence, strategies to reduce recurrence, research needed to ensure treatment meets patients' goals and expectations, alongside improving treatment with lifestyle interventions, pelvic floor muscle training, and pessary management. The group also discussed what research is needed to improve knowledge of the factors influencing the outcome from surgery.

## 2 Identification of Women at High Risk of Prolapse Recurrence and Developing Strategies to Address This

**Figure 1:**
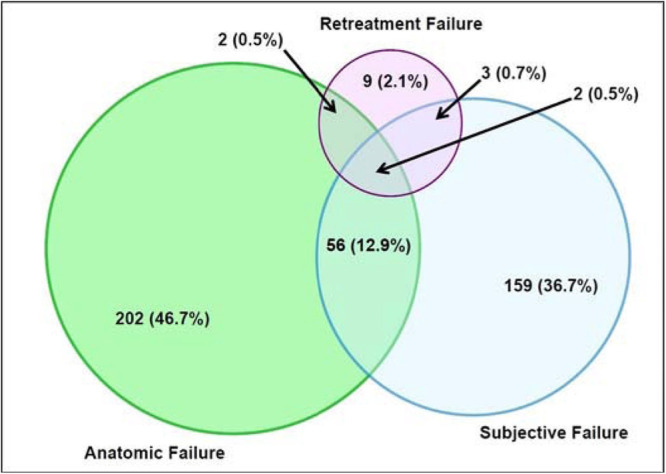
Overlap of surgical outcome definitions at time of surgical failure. Jelovsek JE, Gantz MG, Lukacz E, Sridhar A, Zyczynski H, Harvie HS, Dunivan G, Schaffer J, Sung V, Varner RE, Mazloomdoost D, Barber MD; Eunice Kennedy Shriver National Institute of Child Health and Human Development Pelvic Floor Disorders Network. Success and failure are dynamic, recurrent event states after surgical treatment for pelvic organ prolapse. Am J Obstet Gynecol. 2021 April;224(4):362.e1–362.e11. doi: 10.1016/j.ajog.2020.10.009. Epub 8 October 2020. PMID: 33039390; PMCID: PMC8009767.

Despite considerable endeavour aimed at determining factors which might be associated with successful prolapse repair, research has been hampered by a lack of standardised definition of both surgical and patient‐reported success and awareness of reporting structures that have been developed [5]. Thus, quoted rates of success from prolapse repair vary widely. Where definition of ‘optimal anatomical outcome’ has been equated with cure, this has required perfect anatomical support when measured by validated scoring systems such as the POP quantification system (success defined as stage 0) and satisfactory anatomical outcome, equated with support higher than 1 cm proximal to the hymen. Using these definitions, it has been estimated that 75% of women who present for their annual routine gynaecological examination, with no symptoms of prolapse would fail to meet these outcomes [6]. When applying these two definitions to outcome data from 2‐year follow‐up of women participating in the Colpopexy and Urinary Reduction Efforts (CARE) trial of abdominal sacrocolpopexy, the lowest treatment ‘success’ (19.2%–57.6%) was achieved [7]. In the same trial, when success was defined as the absence of prolapse beyond the hymen (POP‐Q ≥ stage 2) 94.3% achieved surgical success. In terms of patient‐reported success, an absence of ‘awareness of bulge’ symptoms had the strongest relationship with the patients' assessment of overall improvement, treatment success, improvement in symptom bother, and health‐related quality of life. Clearly ‘success’ is a compound variable based upon many factors, anatomical and physiological, intra‐operative, post‐operative, and personal, all of which vary depending upon timing of the procedure with respect to data collection, natural history of the condition and previous treatments, and clinical circumstances (Figure 1). Gynaecologists will often define success as the anatomical outcome, whereas for women the absence of bothersome symptoms, goal attainment, and improved sexual function may be more important markers of success.

In terms of personal factors, those thought to be related to a poorer outcome are a family history of prolapse, the co‐existence of comorbid conditions, the extent of parity, duration of labour and a history of instrumental delivery, and poor pelvic floor strength at initial assessment. There are conflicting data on the impact of older age at time of operation and obesity. Obesity, which is related to morbidity, appears to be mitigated by both prehabilitation and the implementation of evidence informed early recovery after surgery programmes [8, 9, 10, 11, 12, 13].

Identified anatomical factors reported to be associated with a greater likelihood of prolapse recurrence include the presence or absence of levator ani muscle avulsion from the pubis, a larger rather than smaller levator hiatus, and a greater pre‐operative prolapse stage [14]. A 3D study of hiatus and pelvic floor failure showed a complex relationship between levator and genital hiatus areas, length of the pubovisceral and puborectalis muscles and pelvic floor shape in women with prolapse. No single factor explained the progression of pelvic floor failure suggesting, a more complex interaction between these factors [15]. Perhaps unsurprisingly, surgeon experience (experienced vs. inexperienced), inadequate repair technique, a failure to address all compartments of the prolapse, performing a concomitant hysterectomy, and the use of native tissue rather than mesh for repair (in experienced hands), have all been associated with a greater likelihood of either operative failure or an increased likelihood of later prolapse recurrence [16, 17].

From a secondary analysis of data from the randomised multicentre trial PROSPERE, which compared morbidity after prolapse repair with mesh according to the vaginal or laparoscopic approach, factors reported as being related to improvement were an anterior compartment prolapse greater than stage II [OR: 2.93 95% CI (1.22–7.04)], the woman's pre‐operative expectations of improvement in symptoms of bulge [OR: 2.57 95% CI (1.07–6.04)], and the absence of chronic pelvic pain [OR: 4.55 95% CI (1.77–11.46)]. All were statistically significant factors when improved women, defined as a perception of global improvement (PGI‐I) score of much better or better at 1‐year follow‐up, were compared with unimproved women [18].

Post operatively, early resumption of sexual intercourse (before 6 weeks post operatively) is associated with prolapse recurrence [19]. A liberal vs. restrictive approach to the resumption of physical activity and lack of adherence to pelvic floor exercises do not appear to influence the risk of recurrence [20, 21, 22]. Recommendations on the avoidance of abdominal straining, heavy lifting, or management of constipation appear to be based upon custom and practice, rather than any research evidence. In a systematic review of clinical practice guidelines on POP 313 recommendations were examined of which 199 were comparable across guidelines but 31 were found to be unsupported by research evidence [23].

There is limited data on pre‐operative oestrogen therapy. The LOTUS trial showed that a full‐scale trial was feasible but was never funded. The administration of preoperative oestrogen is widely practised, although there is little data to evidence benefit [24].

Finally, there appears to be considerable discordance between the woman's degree of satisfaction with the physician's assessment of the outcome of surgery and surgical success. High failure rates do not appear to be accompanied by high rates of dissatisfaction and reoperation. This mismatch, and that between expectations and goals, may be influenced by inadequate counselling, poor ‘customer’ care, consideration of the impact of prolapse on body image, sexual function, and perhaps other indirect effects; those on a woman's anxiety, depression, and cognitive state [25, 26, 27, 28, 29].

There remains much to do and to learn in terms of personal and postoperative factors which influence the discordance between anatomical and symptomatic results and degree of satisfaction following operative correction for prolapse. Additionally, an agreed outcome dataset and standard ‘timepoint’ for the assessment of ‘cure’ or ‘success’ should be agreed to improve comparability of outcomes. Perhaps this task is impossible, and adoption of the paradigm of person‐centred care, where women are true partners in their care will be the route to ensuring greater success.

## 3 Ensuring Treatment Meets Patient Goals Over Short and Long Term

There are certain ideal attributes to any outcome assessment of treatment. Assessment should be patient‐centred and use realistic and meaningful measure(s) via reliable and reproducible means which can be openly communicated with the patient. Outcomes should be relevant to the patient experience in the short, as well as the long term (> 5 years). Patients often will prioritise relief of symptoms, improvement in daily activities and a return to sexual function as their primary goals to prolapse management. However, unsurprisingly, women seek to avoid post‐operative complications and development of new symptoms or prolapse recurrence as a priority [30]. Patient reported outcome measures (PROMs) have therefore been developed and utilised to capture ‘the most important clinical review of symptom impact and treatment benefit from a patient perspective’ [31].

There is increasing debate on how success or failure of prolapse treatment should be defined from both the clinician's and patient's perspectives as a single entity and whether a recurrent prolapse represents recurring symptoms from the same previously operated site or from a different site and the timescale after index surgery that the prolapse occurred. Reported treatment success can vary from 19.2%–97.2% depending on the definition used [32]. Should we only assess patient symptoms using validated complex PROMs or quality of life assessment tools encompassing multiple facets or by using simpler scales such as the Patients Global Impression of Improvement (PGI‐I)? Should we use even more patient‐friendly reporting of a presence or absence of a single symptom such as a ‘vaginal lump or bulge’ or ‘something coming down’? Although the latter may appear attractive, a patient may have no further prolapse symptoms but be left with debilitating complications from surgery (e.g., pain and dyspareunia). Can we then call that treatment a success? Alternatively, should assessment purely be objective, from information derived from standardised clinical examination? If so, how should failure be defined: prolapse still greater than or equal to stage 2 prolapse, or recurrent prolapse beyond the hymenal ring? However, we know that some women may present with symptomatic prolapse that was never beyond the hymenal ring; this would reach the definition of ‘success’ and would be deemed a success preoperatively, before they had even had treatment. Dynamic MRI has been used to assess prolapse through imaging, but studies suggest that when compared with physical examination there is good to moderate correlation only in the anterior compartment and imaging tends to overestimate the extent of the prolapse [32, 33, 34]. The reference points measured with imaging do not relate to the clinical examination.

Patient‐identified goals of treatment have been recommended as a viable option, with a goal attainment scale then used to determine the patient's reported achievement of those goals. They have been used increasingly over the past 60 years in rehabilitation and mental health treatment [35]. When assessed in women with POP, goal attainment scaling showed women met their goals more often after surgery compared to pessaries in a non‐randomised study [36]. However, this comes with difficulty of setting appropriate, measurable goals and can be time‐consuming. PROMS are also criticised by some because of their subjectivity, despite their being currently ‘mandated’ use in high quality research.

The use of inappropriate metrics has likely led to the overuse of some treatment options and the disregard of others. Ultimately, we need to have a greater understanding of the reasons for treatment failure to improve treatment outcomes. Meaningful outcomes will likely comprise a composite of PROMs, goal achievement, and objective assessment.

## 4 Improving Treatment With Lifestyle Interventions and PFMT

Level 1 evidence exists that lifestyle management limits on‐going development of early POP (stages I, II, and possibly III) [37]. Education improves a woman's knowledge about her POP and assists her to make informed decisions about management.

### 4.1 Reducing Intra‐Abdominal Pressure Rises

There is an association between POP descent and chronic repetitive physically demanding work, such as carrying heavy objects for longer than 2 h per day, more than 10 heavy lifts per day or 20 heavy lifts per week and standing more than 50% of the time [38]. Similarly, management of diseases that increase intra‐abdominal pressure may slow POP progression, for example, constipation causing straining at stool and posterior wall descent, asthma/repeated coughing/COPD inducing repeated descent. Although there is an association between chronic constipation and coughing with the development of prolapse, interventions to treat constipation and reduce coughing to prevent prolapse progression have not been investigated [39]. Although no association between high BMI and development or progression of POP has been demonstrated, 6‐month weight loss can induce significant change and improvement on POP questionnaire scores. These improvements are unexplained by measurements of improvement in genital hiatus, perineal body, or posterior wall positioning, but may relate to reduction in intra‐abdominal pressure.

While vaginal oestrogen does not repair structural pelvic floor defects, oestrogen has a dual effect through changing the quality of epithelium through increased collagen turnover (which may reduce the cross‐linking in immature collagen) and changing sensation as oestrogen acts as a membrane stabiliser to reduce de‐polarisation of neurons. Similarly, pessaries are useful adjuncts to lifestyle management, especially for symptomatic post‐menopausal women [40]. Limiting descent during the day with intervals of anti‐gravity positioning also reduces discomfort.

### 4.2 Optimising Pelvic Floor Muscle Support

Level 1 evidence exists for a dose‐dependent positive impact of PFM training on POP symptoms and associated distress, although there is no evidence that it improves prolapse stage on examination. PFM training is recommended for POP stages I–II (possibly III) [41]. Table 1 summarises key parameters to include in an effective PFM training programme. Whole body training is ineffective for strengthening PFM; in particular, Pilates, core training, and progressive exercises will not induce PFM hypertrophy. Physical activity that supports the perineum during increased intra‐abdominal pressure is adjunctive to PFM training, for example, bike riding, water exercise, and the wearing of support shorts during activities.

**Table 1:** PFM training parameters to improve POP symptoms and stage.

Individual instruction to achieve correct squeeze and elevation of PFM (i.e., not bearing down; confirmed on digital palpation; and independent of inspiration)
Maximal possible contraction
One session of 2–3 sets daily
8–12 repetitions per set
Ten‐second hold of each contraction
Adjunctive lifestyle advice
Adherence strategies to embed behavioural changes
Supervision, re‐assessment, and progression (using valid outcome measures)
Motor learning of ‘the knack’ to precede increases in intra‐abdominal pressure
At least 6 months rehabilitation
Post‐treatment maintenance programme

Changes noted after personalised PFM rehabilitation include reduced levator hiatus size during muscle contraction (although no change at rest); a more circular shape of the levator hiatus associated with generation of greater PFM force; improved POP by one stage; change in sensation of pelvic fullness after activity or at rest; and improved sexual function. Functional pelvic floor bracing with activities of daily living, and optimal defecation techniques become lifelong anticipatory responses to minimise descent. No adverse effects or complications from PFM training have been reported [37].

## 5 Optimising Pessary Care Treatment

There is a long history of women attempting to alleviate prolapse symptoms by inserting items in their vagina. Ancient civilisations used insertion of pomegranates, linen, cotton wool, cork, and brass [42]. Fortunately, modern pessaries are made of materials which are easier to use and, in many cases, enable self‐management.

**Figure 2:**
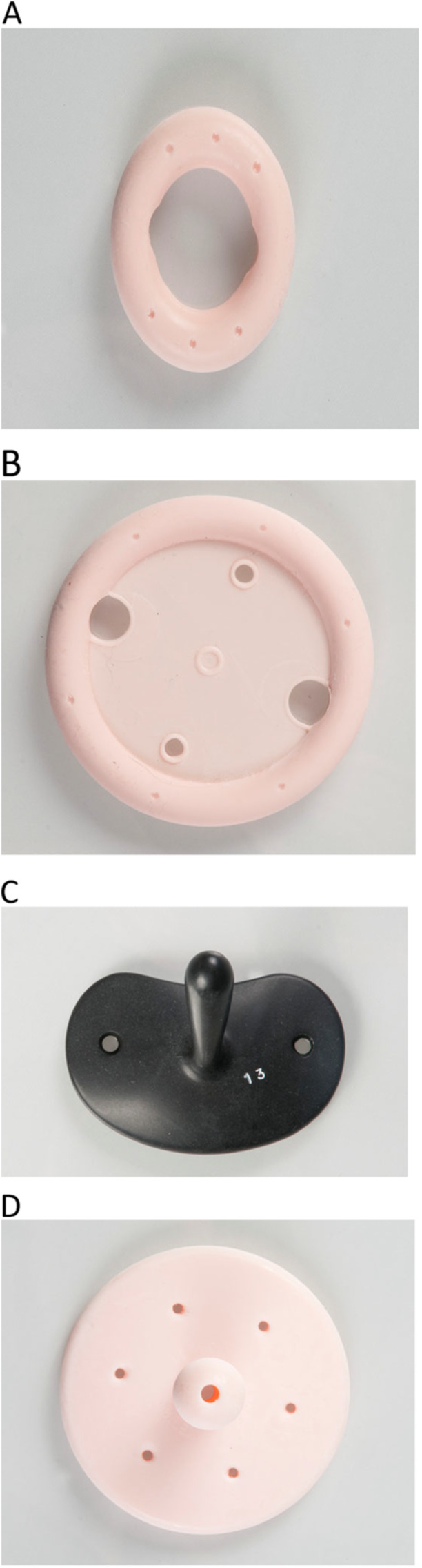
Examples of support ((A) silicone ring and (B) silicone ring with support) and space‐occupying ((C) shelf and (D) gellhorn) pessaries.

The TOPSY trial compared self‐management of pessary for prolapse symptoms with clinic‐based care and reported that women who self‐managed had a similar quality of life but experienced fewer pessary‐associated complications than women who attended a clinic, when assessed at 18 months and at 4 years. There were significant cost savings to the healthcare system with self‐management having an incremental net benefit of £564 UK at 18 months [43, 44]. Women using space‐occupying pessaries (e.g., donuts or cubes) were not included in the TOPSY trial and most women recruited were using a ring or ring with support pessary. In general ring pessaries are used first line as they are easier to change and enable penetrative sexual intercourse, whereas space‐occupying pessaries are harder to change and preclude this (Figure 2). Further research is needed to evaluate if self‐managing a pessary reduces uptake of surgery and if self‐management of space‐occupying pessaries is feasible.

Qualitative data suggest that younger women are more willing to self‐manage and that self‐management is motivated by the reduced number of healthcare visits, increased autonomy, cleanliness and ‘giving their body a break’. There is a lack of correlation between female genital self‐image and willingness to self‐manage. Women who decline self‐management report a lack of confidence, feeling physically unable, wanting clinician‐led care, and a fear of problems or previous problems with a pessary [45]. Women who self‐manage value and feel enabled by a positive relationship with a healthcare provider. Further research into strategies to optimise self‐managed pessary care is needed.

There are very little data on the use of pessaries in younger women and whether or not pessary use for postpartum prolapse symptoms improves pelvic floor recovery for women and leads to anatomical remodelling [46]. The ongoing PEPPY trial is evaluating clinical and cost effectiveness of supervised pelvic floor muscle training plus vaginal pessary compared to supervised pelvic floor muscle training alone for the management of prolapse and is recruiting across the UK. This will provide important information on the use of pessary as an adjunct to PFMT.

Whilst research in pessary provision is ongoing, there is little research on how pessary care should be integrated in the pathway for women presenting with prolapse symptoms and how pessary care could be optimised. All international surveys of pessary practice highlight a common theme of pessary practitioners wanting further training in pessary management and a lack of accreditation for training [47, 48, 49, 50]. The first UK Clinical Guideline for best practice in the use of vaginal pessaries was published in 2021 and outlines treatment algorithms for pessary management [51]. Further materials research is needed to optimise pessary choice to improve fitting success and reduce complications. For example, use of newer 3‐D printed pessaries appears to be associated with improvements in quality of life and in the pelvic floor distress inventory in small pilot studies, warranting further exploration of use [52, 53, 54].

## 6 Improving Outcome From Prolapse Surgery

It is now well‐established that there are significant complications in a proportion of patients when polypropylene mesh (PP) is implanted into the pelvic floor, particularly when used for the treatment of POP. Although PP produces a high level of anatomical cure (90% or more), it is associated with a significant incidence of erosions and other complications, such as continuing pain, leading to dyspareunia [55].

When undergoing cyclical testing, the stress/strain curves for PP show a rightward shift of the hysteresis loop, indicating permanent elongation of the mesh and possible long‐term viscous creep [56].

**Figure 3:**
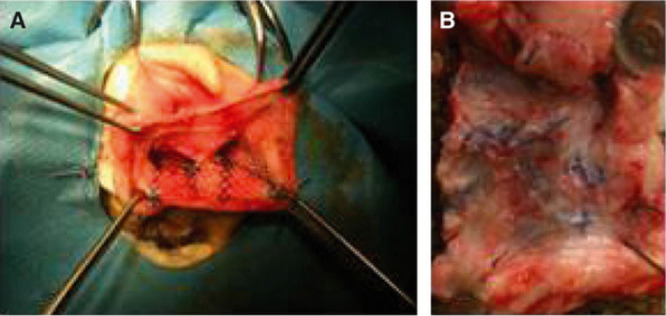
Mesh surgery in sheep. (A) Flat mesh inserted and (B) palpable mesh contraction after 60 days. Manodoro S, Endo M, Uvin P, Albersen M, Vláčil J, Engels A, Schmidt B, De Ridder D, Feola A, Deprest J. Graft‐related complications and biaxial tensiometry following experimental vaginal implantation of flat mesh of variable dimensions. BJOG 2013; 120: 244–250.

In a novel sheep model, PP mesh measuring 50 × 50 mm implanted into the abdominal or vagina of sheep demonstrated contraction, and indeed when implanted into the vagina, exposure of the mesh occurring in 3 out of 10 of the sheep by 90 days (Figure 3) [57].

It is evident that the ideal material should, when implanted, remain relatively elastic to cope with the forces experienced during routine physiological effects, such as coughing, sneezing, and so forth. It should also become stronger at higher strain, like native, healthy fascia, due to remodelling. In the context of POP a patient's own fascia, has poor mechanical properties, and the aim of any implant is to support these tissues.

Unlike an artificial implant, native fascia is not responsible for any significant adverse problems, furthermore the mechanical properties of native fascia are viscoelastic. What is needed in any new biomaterial is to mimic the mechanical properties of native fascia and show tissue integration with it. This has been demonstrated with the use of autologous tissue for the treatment of stress urinary incontinence (SUI). Clearly, in the context of POP and the requirement for larger areas of tissue implant, the use of native fascia implanted into the vagina is more difficult.

Any development of a new biomaterial requires in vitro mechanical testing, in vitro evaluation of cellular integration, in vivo evaluation of the host immune response in animal models, and, specifically, evaluation following implantation into the pelvic floor in an appropriate animal model, such as that described by Manodoro et al. [57].

In evaluating any new biomaterial, it is important to consider the acute inflammatory response mediated by M1 and the chronic remodelling M2 response of tissues. Detailed evaluation of any new biomaterial should be compared to existing materials in an appropriate in vitro model [58].

## 7 Discussion

The Think Tank raised multiple issues that resulted from the perceived inadequacy of surgical treatment of POP. Although there are some national guidelines on the management of prolapse there is insufficient evidence on the effect of lifestyle changes on prolapse progression [59, 60, 61]. Pathways for pessary management are unclear and there is variation on what women are offered [47]. The lack of a strong correlation between symptom bother and examination findings makes it more difficult to assess effectiveness of interventions and to identify tools that can be used to measure cure or improvement. There was consensus on the requirement for further work to develop optimal outcome measures of success. Surgical failure rates are of concern, but so far, synthetic mesh placed vaginally has been associated with high complication rates with insufficient evidence of benefit. Table 2 gives the research questions that need to be answered to improve the management of women with POP.

**Table 2:** Research questions and studies required to answer these questions.

What modifiable personal, operative, postoperative, or rehabilitative factors contribute to discordant anatomical and symptomatic results following operative correction for prolapse? Observational longitudinal cohort studies
At what timepoint should ‘cure’ be assessed? Agreement on standardised terms for use in POP clinical trials
Can we develop a scoring system to guide patients on the risk of recurrence of prolapse after surgery? Development of prediction models from available administrative observational datasets
How does a recurrence of prolapse after repair respond to lifestyle changes? Observational longitudinal propensity matched studies
Should individual tissue profile be considered as a risk factor for no improvement? Observational basic science study
Does large levator hiatus limit gains from lifestyle interventions/PFM strengthening? Potential prospective randomised intervention trials
Does a pessary induce permanent re‐modelling of tissues? Longitudinal imaging and histological studies with appropriate comparator
Does levator avulsion predict failure of pessary? Analyses of administrative datasets with appropriate measures
Does weight gain influence recurrence of POP? Propensity matched cohort studies
Given the lack of prevention studies, is prevention of POP possible? What is the most relevant measure to women: physical exam measures or symptom severity? Qualitative data from women with prolapse
Can we compare goal achievement to patient‐reported outcome measures to understand how success should be defined? Qualitative data from women with prolapse
Can we determine the mechanism of treatment failure to improve outcomes? Analyses of administrative datasets with appropriate measures
What is the optimum pessary material? Comparative prospective RCT with appropriate comparator
Can pessaries be made to fit individual women and optimised to reduce complications? Prospective studies evaluating personalised pessaries with comparator.
Can women self‐manage space‐occupying pessaries? Prospective RCT
Can physical barriers to self‐management be overcome? Qualitative research evaluating barriers
Is there a preventative role for pessary in prolapse treatment? Longitudinal study with comparator group
How best do we manage women with cognitive impairment when using a pessary? Qualitative research evaluating barriers and needs of women with cognitive impairment.
What is the long‐term QOL for women using pessaries compared with surgery? Prospective observational longitudinal study
Is there a continued role for synthetic materials in prolapse surgery? Further animal studies evaluating synthetic materials
New methods to critically evaluate new materials alongside existing PP mesh (fatigue test and SEHI imaging). Should work such as this be mandatory prior to introduction into clinical practice? Further basic science and animal studies evaluating behaviour of synthetic materials in vivo.

## 8 Conclusion

The ICI‐RS 2025 think tank identified important research priorities to reduce prolapse symptom recurrence and treatment failure. Understanding how and when treatment success is assessed is an area requiring further exploration. There is a role for prehab prior to considering surgical intervention since high‐level evidence indicates that physiotherapy and wider conservative management will improve symptoms and limit further descent of lower grades of POP. Further research into vaginal pessaries should focus on understanding if pessaries can aid anatomical remodelling, when they should be offered and if pessary treatment success can be improved with further product development or personalisation. The complications associated with polypropylene insertion should stimulate research into other surgical adjuncts that may improve outcomes without causing serious complications.

## Author Contributions

All authors have made substantial contributions to the conception or design of the work; or the acquisition, analysis, or interpretation of data for the work; Drafting the work or reviewing it critically for important intellectual content; Final approval of the version to be published; and Agreement to be accountable for all aspects of the work in ensuring that questions related to the accuracy or integrity of any part of the work are appropriately investigated and resolved.

## Funding

The authors received no specific funding for this work.

## Ethics Statement

Ethical approval was not required. There is no patient data included and it is not a clinical trial. It reports the findings of a Think Tank held at ICI‐RS 2025.

## Conflicts of Interest

The authors declare no conflicts of interest.

## Data Availability

Data sharing is not applicable to this article, as no new data were created or analysed in this study.
